# Effects of lipid-lowering treatment intensity and adherence on cardiovascular outcomes in patients with a recent myocardial infarction: a Swedish register-based study

**DOI:** 10.48101/ujms.v127.8296

**Published:** 2022-05-04

**Authors:** Maria K. Svensson, Francesc Sorio Vilela, Margrét Leósdóttir, Jonas Banefelt, Maria Lindh, Alexander Rieem Dun, Anna Norhammar, Guillermo Villa

**Affiliations:** a Department of Medical Sciences, Uppsala University, Uppsala, Sweden; b Amgen (Europe) GmbH, Suurstoffi 22, Rotkreuz, Switzerland; c Skåne University Hospital and Department of Clinical Sciences, Lund University, Malmö, Sweden; d Quantify Research, Hantverkargatan 8, Stockholm, Sweden; e Karolinska Institute, Solnavägen 1, Solna, Sweden; f Capio S:t Görans Hospital, Sankt Görans plan 1, Stockholm, Sweden

**Keywords:** Adherence, ezetimibe, statins, treatment intensity, lipid-lowering therapy, major adverse cardiovascular events, myocardial infarction

## Abstract

**Background:**

Oral lipid-lowering treatment (LLT) is the standard of care for patients with cardiovascular disease (CVD). However, insufficient treatment intensity and poor adherence can lead to suboptimal treatment benefit, rendering patients at increased risk of CVD.

**Aims:**

The objective of this study was to evaluate trends in LLT intensity and adherence in Sweden over time, and their association with major adverse cardiovascular events (MACE) after recent myocardial infarction (MI), and also to assess the impact of transition from secondary to primary care on intensity and adherence.

**Methods and results:**

This retrospective observational cohort study used data from Swedish nationwide patient registers and included patients on LLT after an MI in the years 2010–2016 (*n* = 50,298; mean age, 68 years; 69% men). LLT intensity was evaluated over time (overall, for 2010–2013 and for 2014–2016) as the proportion of patients prescribed low-, moderate-, and high-intensity LLT. Adherence was assessed as the proportion of days covered. A combined measure of intensity and adherence was also considered. Differences in treatment patterns and MACE were assessed. Initiation of high-intensity LLT increased over the two time periods studied (2010–2013, 32%; 2014–2016, 91%). Adherence varied by LLT intensity and was highest in patients receiving high-intensity LLT (>80%), especially during the first time period. Little change in treatment intensity or the combined measure of intensity and adherence was observed after transition to primary care. There was a significant association between the combined measure of intensity and adherence and MACE reduction (hazard ratio [95% confidence interval] per 10% increase in the combined measure: 0.84 [0.82–0.86]; *P* < 0.01).

**Conclusion:**

The proportion of post-MI patients with high LLT intensity and adherence has increased in recent years, with little change after transfer from specialist to primary care. The combination of LLT intensity and adherence is important for preventing future cardiovascular events.

## Introduction

Cardiovascular disease (CVD) places a substantial burden on patients and the society, and is the leading cause of morbidity and mortality in Sweden and globally (1–4). High levels of low-density lipoprotein cholesterol (LDL-C) are causal in the development of atherosclerotic CVD (ASCVD) (5, 6), and standard of care for patients with ASCVD, therefore, includes lipid-lowering treatment (LLT) with a statin, with or without ezetimibe (1). In particular, more intensive statin regimens are recommended for patients at a very high risk of CV events, including those who have previously experienced CV events such as myocardial infarction (MI) or ischemic stroke (IS) (1). Lowering of LDL-C to achieve guideline target levels in post-MI patients is proven to reduce CV events, as well as the direct and indirect costs associated with those events (7).

Patients with ASCVD may not receive the full benefit from LLT, either because their treatment regimen is not sufficiently intensive or because their adherence is poor (8–10). While studies have shown that poor adherence to LLT is associated with an increase in CV outcomes (11, 12), there are limited data on interactions between treatment intensity and adherence. Furthermore, the impact on lipid management when transitioning from secondary to primary care (in Sweden, usually 12–52 weeks after an MI) is unknown, although the use of LLT and adherence to prescribed regimens in primary care have been reported to be low (13–16).

This study aimed to evaluate trends in LLT intensity and adherence in Sweden over time, and their association with outcomes in patients who had previously experienced an MI and initiated LLT. In patients who transitioned from secondary to primary care during the study, LLT intensity and adherence were assessed before and after transition.

## Methods

### Study design and patient population

**Figure 1:**
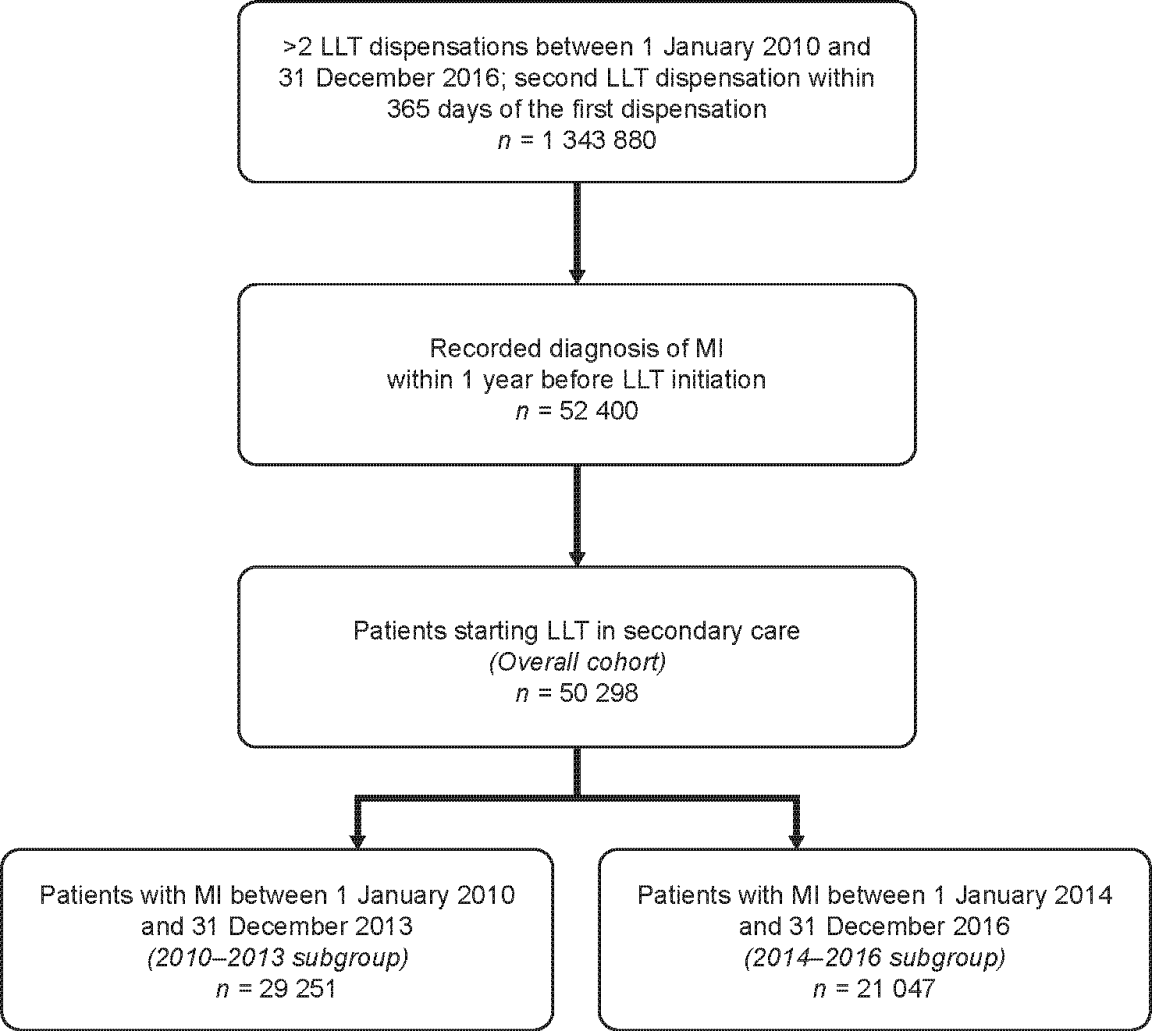
Identification of patients in the overall population and subgroups. LLT: lipid-lowering therapy; MI: myocardial infarction.

This was a retrospective observational cohort study using data from Swedish nationwide patient registers (17, 18). Patient selection is shown in Figure 1. Eligible patients were adults (age ≥ 18 years) at the time of initiation of LLT who had experienced a recent MI (within 365 days before initiating LLT) and were followed up for the occurrence of subsequent major adverse CV events (MACE; MI, IS, or CV death) until December 2017. MI was defined on the basis of both the primary and secondary diagnoses (International Classification of Diseases, Tenth Revision, Clinical Modification [ICD]-10 code). Initiation of LLT was defined as at least two dispensations for a statin, with or without ezetimibe (19), with the second dispensation made between 1 January 2010 and 31 December 2016 (the index date), and within 365 days of their first dispensation. This definition of two or more dispensations of a statin (with or without ezetimibe) within 365 days of the first dispensation was to ensure an adequate time period for patients to have received a second dispensation, and therefore, still be on treatment. Patients identified as having received LLT before the index MI were excluded from the analysis.

The baseline period for assessment of demographics and comorbidities for each patient was defined as the period between the earliest date in the register database (1 January 2001) and the index date (i.e. the date of the second dispensation). Two subgroups were defined based on the year of MI diagnosis (2010–2013 vs. 2014–2016) to evaluate the impact of changes in LLT recommendations due to the 2012 update in the European guidelines on CVD prevention (20), the introduction of generic atorvastatin in 2013, and the 2014 treatment recommendations on the prevention of ASCVD published by the Swedish Medical Product Agency (21).

Ethical approval was obtained from the Swedish ethical review board in Stockholm on 11 April 2018 (reference number 2018/610-31). Only pseudonymized data were retrieved and analyzed.

### Data sources

Patient-level data from the National Patient Register (NPR), Prescribed Drug Register (PDR), and Cause of Death Register (CDR) were linked by the Swedish National Board of Health and Welfare using unique personal identifiers. The Swedish NPR contains information on all hospital stays at public hospitals and all outpatient specialist visits, including discharge diagnoses (according to the ICD – 10 code) and procedures (coded according to the Swedish Classification of Care Measures [KVÅ]). The PDR includes data on all dispensations from pharmacies, including drug type, dispensing date, dose, and pack size, as well as speciality, and workplace of prescribing physician. The CDR includes confirmed dates and causes of death. Deaths were considered to be cardiovascular if any ICD-10-CM code from I00 to I78 was recorded. Patients with dispensations of any statin, with or without ezetimibe, were identified in the PDR for potential inclusion in the study. Differentiation between primary care and secondary care settings was based on the workplace code of the prescribing physician of each patient’s dispended prescription (see details below).

### Definitions

LLT intensity was evaluated over time in terms of the proportions of patients receiving low-, moderate-, and high-intensity LLT, based on the American College of Cardiology/American Heart Association (ACC/AHA) guidelines for cholesterol treatment (Supplementary Table 1) (22). Ezetimibe monotherapy was considered low intensity, and combination therapy with statins considered high intensity, consistent with the approach used in the EUROASPIRE study (23). Individuals switching intensity groups during the year were assigned to the category with the longest duration during the year.

*Treatment adherence* was based on LLT dispensation data and assessed as the proportion of days covered (PDC; that is, the ratio of the number of days for which the patient has been prescribed medication to the number of days on which they are eligible for the medication), where the length of each prescription was calculated based on the quantity dispensed and the assumption that patients were prescribed one tablet daily. When prescriptions were overlapping, the calculation of supply restarted from each new filled dispensation, discarding the remaining supply from the previous dispensation. When analysing the association of adherence and outcomes, patients were considered as untreated in any given year during which they did not fill any LLT dispensations.

A *combined measure of treatment intensity and adherence* (defined as the continuous time-weighted average treatment over each year, according to the definitions in Supplementary Table 2) was estimated and used to capture the individual treatment intensity and adherence covariates.

To evaluate *transition from secondary to primary care*, treatment was compared and assessed during the 1 year before and after transition. Transition to primary care from secondary care was defined based on the information directly available in the source data on whether the dispensed prescription was made in primary care or in secondary care (the prescribing physician’s workplace code). Patients initially prescribed LLT by a physician in secondary care were assumed to have transitioned to primary care once they dispensed a prescription of LLT made in primary care.

### Statistical analysis

Demographics and baseline characteristics were presented descriptively using proportions or percentages for categorical variables and with mean and standard deviation for continuous variables.

Intensity was analyzed as a categorical variable in the descriptive analyses and as a continuous variable (time-weighted average treatment over each year) in the time-to-event analyses based on the estimated percentage reduction in LDL-C (Supplementary Table 2). Data were assessed for each year of follow-up.

Adherence was analyzed as a continuous variable (0–100%) and was also categorized for the descriptive part of the study (adherent = PDC ≥ 80%). Adherence was calculated and updated annually (i.e. as a time-varying exposure).

The association between MACE and the combined measure of treatment intensity and adherence was estimated using Cox proportional hazards regression models by time-varying exposures for the combined measure, updated at annual intervals. Age was used as the timescale to minimize any association between the event rate and time. The proportional hazards assumption was tested for all Cox regression models using tests based on the Schoenfeld residuals. If the tests indicated that the proportional hazards assumption did not hold, selected variables were incorporated as stratification variables to allow the baseline hazard to vary across strata. If the combined measure of treatment intensity and adherence displayed non-proportionality of hazards after stratification of appropriate covariates, the length of follow-up was limited by 1 year at a time. As adherence, treatment intensity, and the combined measure were estimated annually, they were all modelled using a 1‑year lag (i.e. adherence and treatment intensity in a year were used to estimate the risk of CV events in the subsequent year).

The predicted reduction in CV risk was estimated using the combined measure of treatment intensity and adherence for the overall patient cohort using information from the Cox proportional hazards regression models (in which patients’ average intensity was calculated and used in the Cox regression models to predict hazard ratios [HRs]) *within six patient groups*: non-adherent patients (PDC <80%) receiving low-, moderate-, and high-intensity LLT, and adherent patients (PDC ≥ 80%) receiving low-, moderate-, and high-intensity LLT.

To distinguish incident CV events from other events (e.g. prevalent CVD events, re-visits, and transfers), only the primary diagnoses with accompanying hospitalizations (i.e. diagnoses made in the inpatient setting) were considered new CV events. In addition, hospitalizations for which there was an accompanying procedure code for either intravenous administration or pacemaker adjustments with a maximum hospitalization stay of 1 day were not considered as new CV events.

Transition from secondary care to primary care (first transition for each patient) was analyzed descriptively, with differences in treatment intensity and adherence assessed during the 365 days before and after transition.

Data management and statistical analyses were performed using MySQL and Stata 16 (StataCorp LP, College Station, TX, USA).

## Results

### Patient population, treatment intensity, and adherence

In total, 50,298 patients with LLT initiation within 1 year after their MI were included in the analysis, with 29,251 patients in the 2010–2013 subgroup, and 21,047 patients in the 2014–2016 subgroup (Figure 1). Patients in the overall cohort had a mean age of 68 years, approximately 70% were men, and 57% of patients overall were initiated on high-intensity statins (Table 1). Baseline characteristics were similar in each of the two time periods, except for statin use, with more than 90% of patients in the 2014–2016 subgroup being initiated on high-intensity statins and 8% on moderate-intensity statins, compared with 32 and 68%, respectively, in the 2010–2013 subgroup. Fewer than 1% of patients were initiated on ezetimibe with or without a statin. The median time between the first and the second dispensation was 84 days, aligned with a typical prescription pattern in Sweden.

**Table 1:** Baseline clinical characteristics of study participants.

Variables	Overall cohort *n* = 50,298	2010–2013 subgroup *n* = 29,251	2014–2016 subgroup *n* = 21,047
Age (years), mean (SD)	67.9 (12.3)	68.0 (12.3)	67.7 (12.2)
Male sex	34,933 (69.5)	20,181 (69.0)	14,752 (70.1)
Duration of follow-up (years), mean (SD)	4.4 (2.1)	5.6 (1.9)	2.9 (0.9)
Charlson comorbidity index			
1	34,595 (68.8)	20,003 (68.4)	14,592 (69.3)
2+	15,703 (31.2)	9,248 (31.6)	6,455 (30.7)
CV history			
Coronary revascularization procedure	36,765 (73.1)	20,688 (70.7)	16,077 (76.4)
Unstable angina	9,171 (18.2)	5,837 (20.0)	3,334 (15.8)
IS	1,464 (2.9)	962 (3.3)	502 (2.4)
Peripheral artery disease	918 (1.8)	605 (2.1)	313 (1.5)
Transient ischemic attack	851 (1.7)	553 (1.9)	298 (1.4)
Abdominal aortic aneurysm	377 (0.8)	219 (0.8)	158 (0.8)
CV risk factors			
Antithrombotic drug use*	47,336 (94.1)	27,346 (93.5)	19,990 (95.0)
Antihypertensive drug use†	44,916 (89.3)	26,138 (89.4)	18,778 (89.2)
Atrial fibrillation	6,063 (12.1)	3,615 (12.4)	2,448 (11.6)
Type 2 diabetes	5,891 (11.7)	3,485 (11.9)	2,406 (11.4)
CKD stages 4–5	360 (0.7)	165 (0.6)	195 (0.9)
Carotid stenosis	179 (0.4)	122 (0.4)	57 (0.3)
Initial LLT dispensation			
High-intensity statin	28,475 (56.6)	9,264 (31.7)	19,210 (91.3)
Moderate-intensity statin	21,543 (42.8)	19,799 (67.7)	1,744 (8.3)
Low-intensity statin	170 (0.3)	148 (0.5)	22 (0.1)
Statin with ezetimibe	78 (0.2)	26 (0.1)	52 (0.3)
Ezetimibe monotherapy	32 (0.1)	14 (0.1)	18 (0.1)

CKD: chronic kidney disease; CV: cardiovascular; IS: ischemic stroke; LLT: lipid-lowering therapy; SD: standard deviation.

Note: Data are *n* (%) unless stated otherwise.

* Refers to anti-thrombotic drug use within the last year.

† Refers to anti-hypertensive drug use within the last year.

**Figure 2:**
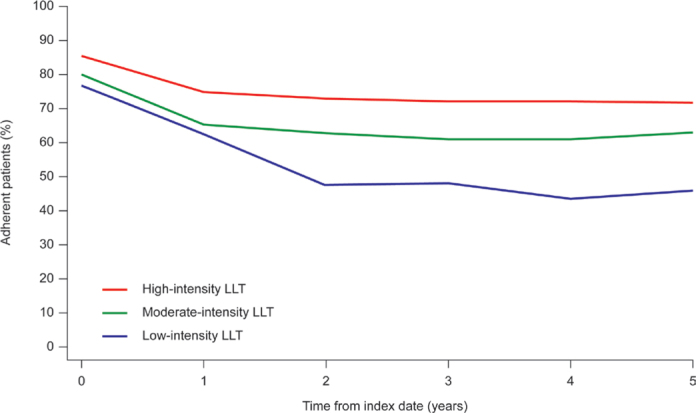
Percentage of adherent patients over time, classified by treatment intensity, in the overall cohort. Adherent patients defined as those with PDC ≥ 0.8. LLT: lipid-lowering therapy; PDC: proportion of days covered.

In the overall patient cohort, PDC varied by treatment intensity, with the highest adherence in patients receiving high-intensity LLT (Figure 2). Adherence decreased over time, with the greatest decrease observed in the first year of follow-up.

**Figure 3:**
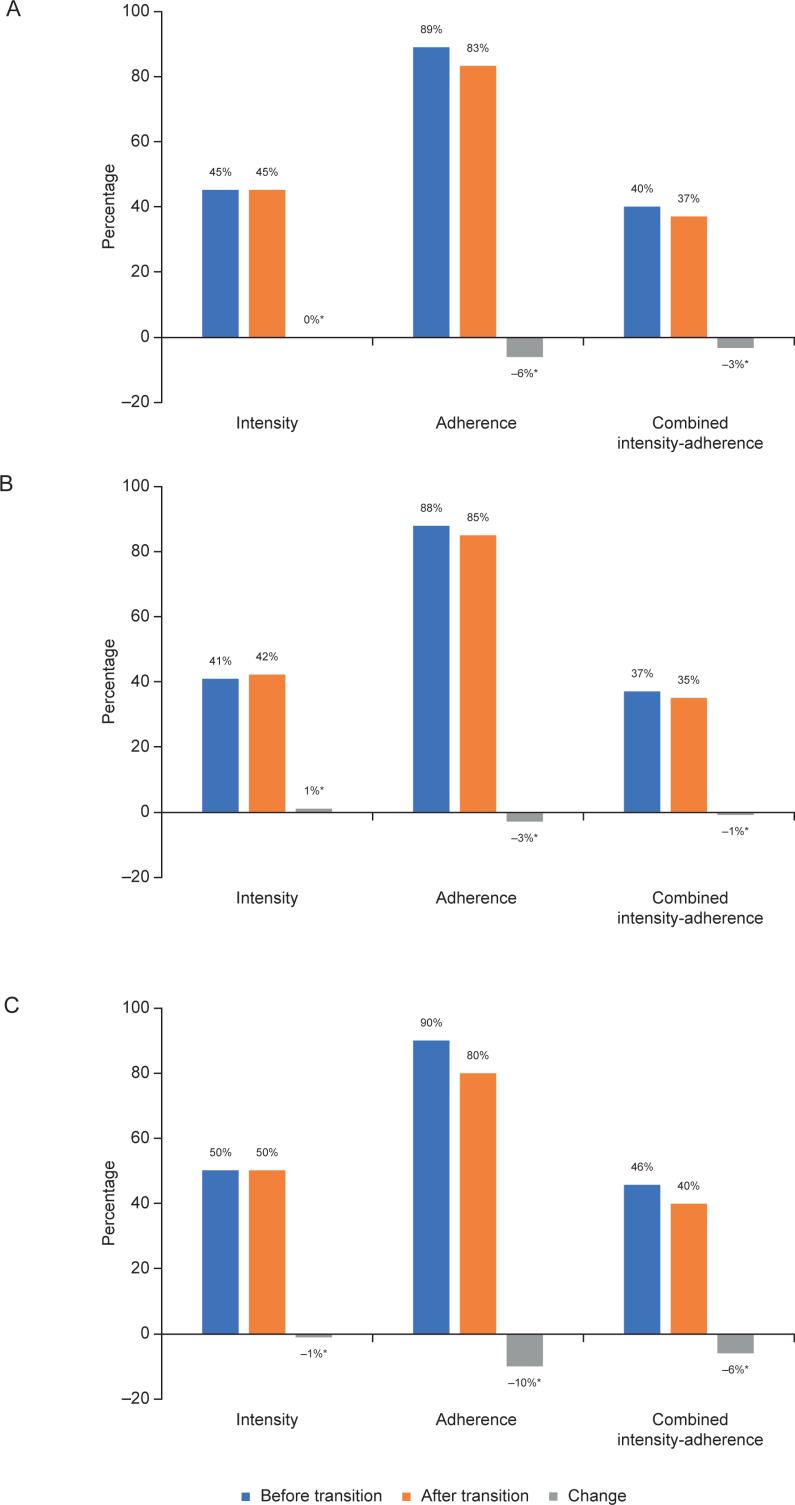
Treatment intensity, adherence, and the combined measure of treatment intensity and adherence before and after transition from secondary care to primary care in (a) the overall cohort, (b) the 2010–2013 subgroup, and (c) the 2014–2016 subgroup.

Assessment of treatment patterns before and after transition from secondary to primary care showed that PDC decreased from 89 to 83% in the overall cohort (*n* = 40,999) (Figure 3). The change was greater in the 2014–2016 subgroup (from 90 to 80%; *n* = 15,804) than in the 2010–2013 subgroup (from 88 to 85%; *n* = 25,195). Changes in treatment intensity and in the combined measure of treatment intensity and adherence after transition were numerically small (Figure 3). When comparing the last prescription dispensed before transition to the first prescription dispensed after transition, mean intensity was 49% both before and after transition, in both the 2010–2013 subgroup and the 2014–2016 subgroup.

### Association of intensity and adherence with CV events

**Figure 4:**
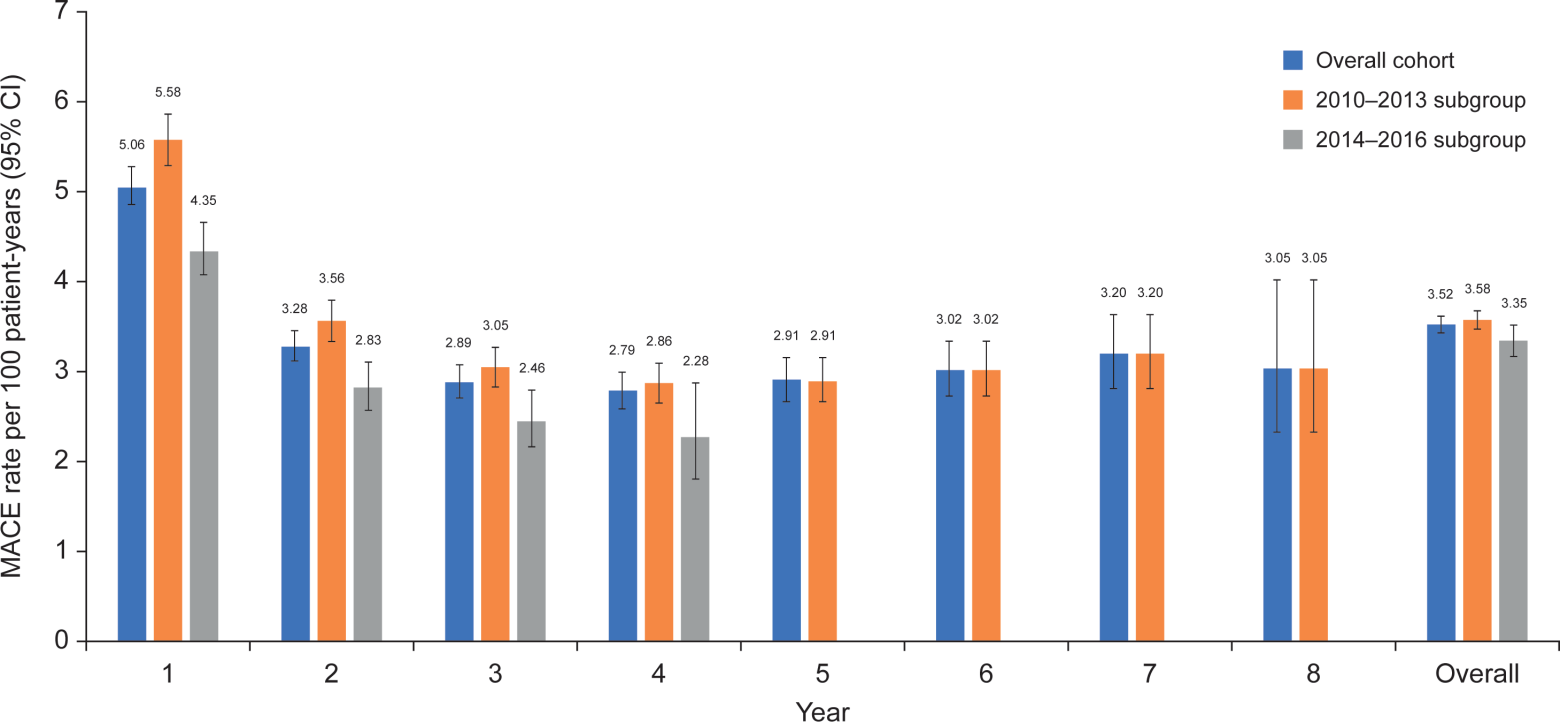
Annual MACE rates. Maximum follow-up in the overall population and the 2010–2013 subgroup was 8 years; maximum follow-up in the 2014–2016 subgroup was 4 years. CI: confidence interval; MACE: major adverse cardiovascular events.

The overall incidence of MACE and individual CV events is shown in Supplementary Table 3. Annual MACE rates tended to decrease in the first 4 years of follow-up and were lower in the year 2014–2016 subgroup than in the 2010–2013 subgroup (Figure 4). In the multivariable Cox regression analysis, there was a significant association between the combined measure of treatment intensity and adherence and MACE (Table 2). In the overall population, an increase of 10 percentage points in the combined measure resulted in a reduction in the risk of MACE (HR: 0.84 (95% confidence interval [CI]: 0.82–0.86; *P* < 0.01); HRs for the 2010–2013 and 2014–2016 subgroups were 0.84 (95%CI: 0.82–0.87; *P* < 0.01) and 0.87 (95%CI: 0.83–0.92; *P* < 0.01), respectively). Similar results were obtained with Cox regression analyses incorporating intensity and adherence separately, as well as the combined measure (Supplementary Table 4) and incorporating intensity and adherence but not the combined measure (Supplementary Table 5).

**Table 2:** Multivariable Cox regression analysis of association between the combined measure of treatment intensity and adherence and MACE.

Variable	Overall cohort	2010–2013 subgroup	2014–2016 subgroup
10% increase in combined intensity-adherence	0.84 (0.82–0.86)*	0.84 (0.82–0.87)*	0.87 (0.83–0.92)*

CI: confidence interval; CKD: chronic kidney disease; HR: hazard ratio; LLT: lipid-lowering therapy; MACE: major cardiovascular events.

Note: Values are expressed as HR (95% CI). Model adjusted for the following covariates: initial use of high-intensity LLT; sex; hypertension; CKD stages 4–5; diabetes; Charlson comorbidity index; atrial fibrillation; year of follow-up. In addition, the model uses age as the time scale to control for age. The model incorporates stratification variables rather than covariates as necessary to handle issues related to non-proportionality of hazards. The length of follow-up was limited to 4 years to handle non-proportional hazards.

* *P* < 0.01.

**Figure 5:**
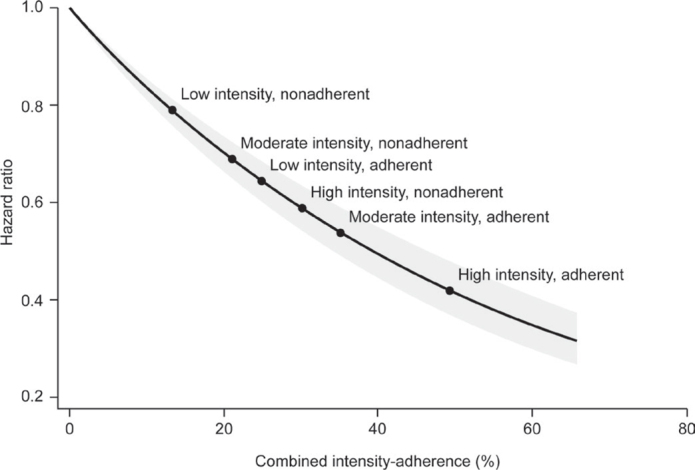
Predicted CV risk reduction using the combined measure of treatment intensity and adherence for the overall patient cohort. CV: cardiovascular.

The association between the combined measure and outcomes was also assessed in the six prespecified patient categories in the overall patient cohort (Figure 5). Adherent patients receiving high-intensity LLT had the lowest MACE risk compared with those who were untreated for more than 1 year, followed by adherent patients receiving moderate-intensity LLT (Figure 5; Supplementary Figure 1). The smallest reduction in MACE risk was seen in non-adherent patients receiving low- or moderate-intensity LLT.

## Discussion

This study enrolled patients who had previously experienced a recent MI and initiated LLT within a year after the acute event. More than 90% of patients in the year 2014–2016 subgroup initiated high-intensity LLT compared with only one-third of patients in the 2010–2013 subgroup. This striking difference is consistent with guidance in the updated treatment recommendations on prevention of ASCVD published by the Swedish Medical Product Agency in 2014, and European Society of Cardiology guidelines published in the preceding years, recommending more intensive statin treatment in patients with established coronary artery disease (21, 24, 25). Patients in the 2014–2016 subgroup more frequently received high-intensity LLT and had lower annual MACE rates than those in the 2010–2013 subgroup, suggesting that increased adherence to the updated treatment guidelines contributed to reduced risk of CV events using more intensive statin regimens.

Levels of adherence were higher in patients initiating high-intensity LLT than in those initiating moderate- or low-intensity LLT but tended to decrease over time in all groups. Patients initiating low-intensity LLT were few in number and probably represent a subpopulation that differs from the overall population in terms of increased frailty and more comorbidities (Supplementary Table 6). In this analysis, treatment intensity was maintained after transition from secondary care to primary care. While there was a slight decrease in adherence during the first year, it cannot be determined whether this was the result of transition or due to other factors such as declining adherence with long-term treatment.

Analysis of the combined measure of treatment intensity and adherence showed a significant association with MACE, with the lowest risk of MACE among patients with a combination of high adherence and high-intensity LLT, and the highest risk in non-adherent patients with low-intensity LLT. This supports that adherence, in addition to LLT intensity, is important for preventing MACE.

Our results from Sweden are consistent with those of the previous study from the UK, which included patients with a first LLT dispensation between 2010 and 2013 (26). In a subgroup of patients with documented ASCVD, adherence also decreased over time, and patients receiving high-intensity LLT were more likely to be adherent than those receiving low-intensity LLT. Using a combined measure of treatment intensity and adherence, there was an association with both LDL-C reduction and CV outcomes. As in this study, adherent patients receiving high-intensity LLT had the lowest risk of subsequent CV events. Similar data have also been reported from Germany and France (27, 28). Importantly, patients who have previously experienced CV events such as MI or IS are known to be at a very high risk of subsequent events (29), and early up-titration to high-intensity LLT can reduce the risk (30).

One of the main strengths of this study is the high degree of validity, completeness, and data quality in the Swedish registers, which enables reliable real-world data analyses. Moreover, the study contains a large patient cohort, with a long duration of follow-up. Overall, data were available from 1 January 2001 to 31 December 2017, so ending the patient inclusion period on 31 December 2016 ensured at least 1 year of follow-up for each patient and enabled results to be based on recent data while also providing a sufficiently large study sample. It also allowed the impact of changes to the European guidelines and the introduction of generic atorvastatin in Europe (February 2012) to be assessed. Limitations of the study include the lack of laboratory data, particularly for LDL-C and other lipid parameters. In addition, results for patients initiating low-intensity LLT may be biased, as noted above, as patients who initiate treatment on low-intensity LLT are likely to have done so because of comorbidities and higher risk of side-effects. It should also be noted that intensity, adherence, and the combined measure were all modelled using a 1-year lag. This was done to ensure stable estimates of PDC and the correct temporal order between the exposure and the outcome. However, the 1-year lag may lead to an underestimation of the benefits of high-intensity LLT and adherence to treatment on the risk of MACE, potentially making our estimates conservative. In the analysis of transition from secondary to primary care, only the first transition was evaluated, and differences in intensity and adherence were described only in the 1 year before and 1 year after transition. Further research is, therefore, needed to describe potential changes in treatment patterns beyond the first year after care setting transitions.

In conclusion, the intensity of initial LLT in patients who experience an MI has increased over time, with most patients initiating LLT between 2014 and 2016 doing so with high-intensity LLT, in accordance with current treatment recommendations at the time. Achieving a combination of high-intensity LLT and high adherence was associated with the lowest risk of subsequent MACE. This highlights the importance of initiating treatment with high-intensity LLT in a very high-risk patients and promoting treatment adherence with appropriate measures. In patients who transition from secondary to primary care in Sweden, treatment intensity and adherence are generally well maintained during the first year after transition.

## Supplementary Material

Effects of lipid-lowering treatment intensity and adherence on cardiovascular outcomes in patients with a recent myocardial infarction: a Swedish register-based study

## Data Availability

The data underlying this article are available in the article and in its online supplementary material.
