# Supplementary material for: Effects of lipid-lowering treatment intensity and adherence on cardiovascular outcomes in patients with a recent myocardial infarction: a Swedish register-based study
Source: Ups J Med Sci. 2022 May 4;127:10.48101/ujms.v127.8296. doi: 10.48101/ujms.v127.8296 (PMC9171571; doi:10.48101/ujms.v127.8296)

Supplementary Data – Svensson et al.

Effects of lipid-lowering treatment intensity and adherence on cardiovascular outcomes in patients with a recent myocardial infarction: a Swedish register-based study

Supplementary tables

Table S1 Definition of low, moderate, and high-intensity LLT*

Intensity	LDL-C reduction	Regimens
Low	<30%	Fluvastatin 20 or 40 mg; pravastatin 5, 10, or 20 mg; simvastatin 10 mg
Moderate	30–49%	Fluvastatin 80 mg; simvastatin 20 or 40 mg; atorvastatin 10 or 20 mg; pravastatin 40 or 80 mg; lovastatin 40 mg; rosuvastatin 5 or 10 mg
High	≥50%	Atorvastatin 40 or 80 mg; rosuvastatin 20 or 40 mg; simvastatin 80 mg

*Based on the American College of Cardiology/American Heart Association (ACC/AHA) guidelines for cholesterol treatment (Stone NJ, et al. Circulation 2014;129:S1–45). LDL-C, low-density lipoprotein cholesterol; LLT, lipid-lowering therapy.

Table S2 Percentage reduction in LDL-C by drug type and dose

	Dose (mg/day)				
**LLT**	5	10	20	40	80
Fluvastatin (1)	–	–	21	27	33
Pravastatin (1)	–	20	24	29	–
Simvastatin (1)	–	27	32	37	42
Atorvastatin (1)	–	37	43	49	55
Rosuvastatin (1)	38	43	48	53	–
Ezetimibe monotherapy (2,3)	–	19	–	–	–
Ezetimibe added to statin* (3,4)	–	24	–	–	–

*Incremental 24% LDL-C reduction calculated after the statin reduction is approximately the same as an additional ~10–15% reduction from pre-treatment LDL-C. For example, the expected reduction in LDL-C with atorvastatin 20 mg is 43%, and the additional reduction with ezetimibe is 14% (i.e. 24% of 57%), leading to a total LDL-C reduction of 57%. The expected reduction with atorvastatin 80 mg is 55%, and the additional reduction with ezetimibe is 11% (i.e. 24% of 45%), leading to a total reduction of 66%. LDL-C, low-density lipoprotein cholesterol; LLT, lipid-lowering therapy.

References

1. National Institute for Health and Care Excellence (NICE). Cardiovascular disease: risk assessment and reduction, including lipid modification. Clinical guideline [CG181]. 2014. Available from: https://www.nice.org.uk/guidance/cg181

2. Pandor A, Ara RM, Tumur I, Wilkinson AJ, Paisley S, Duenas A, et al. Ezetimibe monotherapy for cholesterol lowering in 2,722 people: systematic review and meta-analysis of randomized controlled trials. J Intern Med. 2009;265:568-580. doi: 10.1111/j.1365-2796.2008.02062.x

3. Ara R, Tumur I, Pandor A, Duenas A, Williams R, Wilkinson A, et al. Ezetimibe for the treatment of hypercholesterolaemia: a systematic review and economic evaluation. Health Technol Assess. 2008;12:iii, xi-xiii, 1-212. doi: 10.3310/hta12210

4. Cannon CP, Blazing MA, Giugliano RP, McCagg A, White JA, Theroux P, et al. Ezetimibe Added to Statin Therapy after Acute Coronary Syndromes. N Engl J Med. 2015;372:2387-2397. doi: 10.1056/NEJMoa1410489

Table S3 Distribution of MACE during follow-up

	**Overall cohort** ***n* = 50,298**	**2010–2013 subgroup** ***n* = 29,251**	**2014–2016 subgroup** ***n* = 21,047**
MACE, n	6,922	5,262	1,660
Hospitalization for MI (non-fatal), n (%)	3,289 (48)	2,444 (46)	845 (51)
Hospitalization for IS (non-fatal), n (%)	1,292 (19)	1,021 (19)	271 (16)
CV death, n (%)	2,341 (34)	1,797 (34)	544 (33)

CV, cardiovascular; IS, ischemic stroke; MACE, major adverse cardiovascular events; MI, myocardial infarction.

Table S4 Multivariable Cox regression analysis of association between intensity, adherence, and the combined measure of treatment intensity and adherence, and MACE

Variable	Overall cohort	2010–2013 subgroup	2014–2016 subgroup
10% increase in intensity	1.06 (1.02–1.11)**	1.10 (1.05–1.16)**	1.03 (0.94–1.13)
10% increase in adherence	1.06 (1.03–1.10)**	1.03 (0.99–1.07)	1.07 (0.99–1.14)
10% increase in combined intensity-adherence	0.71 (0.65–0.77)**	0.74 (0.67–0.81)**	0.77 (0.65–0.90)**

Values are expressed as HR (95% CI). Models adjusted for the following covariates: initial use of high-intensity LLT; sex; hypertension; CKD stages 4–5; diabetes; Charlson comorbidity index; atrial fibrillation; year of follow-up. Additionally, the models use age as the time scale to control for age. The model incorporates stratification variables rather than covariates as necessary to handle issues related to non-proportionality of hazards. Length of follow-up was limited to 4 years to handle non-proportional hazards. **P < 0.01; *P < 0.05; CI, confidence interval; CKD, chronic kidney disease; HR, hazard ratio; LLT, lipid-lowering therapy; MACE, major cardiovascular events

Table S5 Multivariable Cox regression analysis of association between intensity and adherence, and MACE

Variable	Overall cohort	2010–2013 subgroup	2014–2016 subgroup
10% increase in intensity	0.98 (0.94–1.02)	1.01 (0.97–1.06)	0.95 (0.88–1.03)
10% increase in adherence	0.94 (0.93–0.96)**	0.93 (0.92–0.95)**	0.96 (0.93–0.99)**

Values are expressed as HR (95% CI). Models adjusted for the following covariates: initial use of high-intensity LLT; sex; hypertension; CKD stages 4–5; diabetes; Charlson comorbidity index; atrial fibrillation; year of follow-up. Additionally, the models use age as the time scale to control for age. The model incorporates stratification variables rather than covariates as necessary to handle issues related to non-proportionality of hazards. Length of follow-up was limited to 4 years to handle non-proportional hazards. **$P < 0.01$; *$P < 0.05$; CI, confidence interval; CKD, chronic kidney disease; HR, hazard ratio; LLT, lipid-lowering therapy; MACE, major cardiovascular events

Table S6: Baseline characteristics by initial use of statin intensity

	**Low-intensity statins**<br>***n* = 170**	**Moderate-intensity statins**<br>***n* = 21,543**	**High-intensity statins**<br>***n* = 28,475**
Duration of follow-up (years), mean (SD)	4.5 (2.5)	5.5 (2.17)	3.6 (1.5)
Age (years), mean (SD)	77.0 (13.7)	69.5 (12.6)	66.7 (11.8)
Charlson comorbidity index, n (%)			
1	83 (48.9)	14,048 (65.2)	20,387 (71.6)
2+	87 (51.2)	7,495 (34.8)	8,088 (28.4)

Supplementary figures

Figure S1 Predicted cardiovascular risk reduction using the combined measure of treatment intensity and adherence for a) the 2010–2013 and b) 2014–2016 subgroups

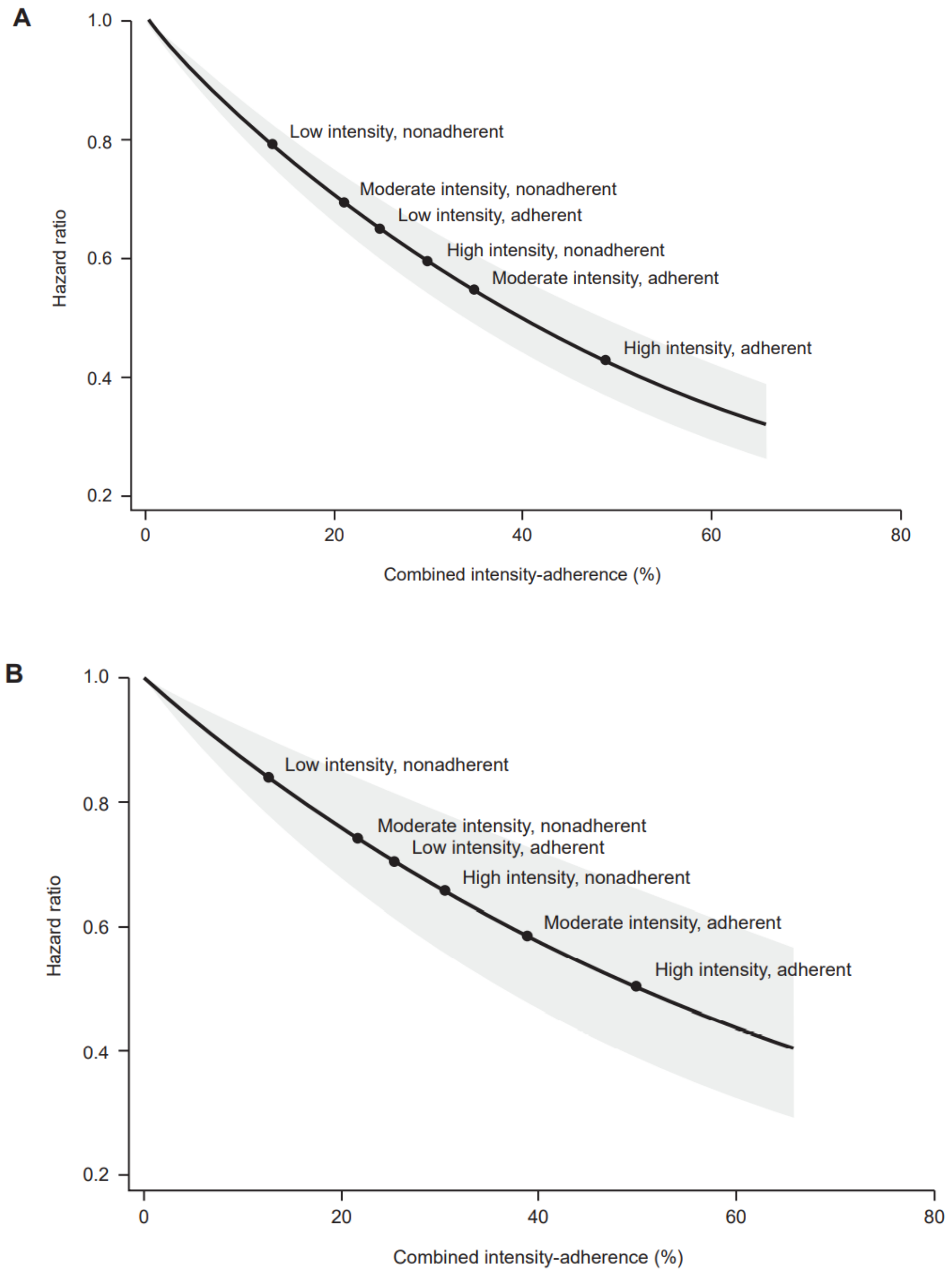

Supplement: Effects of lipid-lowering treatment intensity and adherence on cardiovascular outcomes in patients with a recent myocardial infarction: a Swedish register-based study [file UJMS-127-8296-s001.pdf]
